# Changes in parasite traits, rather than intensity, affect the dynamics of infection under external perturbation

**DOI:** 10.1371/journal.pcbi.1006167

**Published:** 2018-06-11

**Authors:** Suma Ghosh, Matthew J. Ferrari, Ashutosh K. Pathak, Isabella M. Cattadori

**Affiliations:** 1 Department of Mathematics, School of Natural Sciences, Shiv Nadar University, Dadri, Uttar Pradesh, India; 2 Center for Infectious Disease Dynamics, The Pennsylvania State University, State College, Pennsylvania, United States of America; 3 Odum School of Ecology, The University of Georgia, Athens, Georgia, United States of America; CNRS, FRANCE

## Abstract

Understanding the mechanisms that generate complex host-parasite interactions, and how they contribute to variation between and within hosts, is important for predicting risk of infection and transmission, and for developing more effective interventions based on parasite properties. We used the *T. retortaeformis* (TR)-rabbit system and developed a state-space mathematical framework to capture the variation in intensity of infection and egg shedding in hosts infected weekly, then treated with an anthelminthic and subsequently re-challenged following the same infection regime. Experimental infections indicate that parasite intensity accumulates more slowly in the post-anthelminthic phase but reaches similar maximum numbers. By contrast, parasite EPG (eggs per gram of feces) shed from rabbits in the post-treatment phase is lower and less variable through time. Inference based on EPG alone suggests a decline in parasite intensity over time. Using a state-space model and incorporating all sources of cross-sectional and longitudinal data, we show that while parasite intensity remains relatively constant in both experimental phases, shedding of eggs into the environment is increasingly limited through changes in parasite growth. We suggest that host immunity directly modulates both the accumulation and the growth of the parasite, and indirectly affects transmission by limiting parasite length and thus fecundity. This study provides a better understanding of how within-host trophic interactions influence different components of a helminth population. It also suggests that heterogeneity in parasite traits should be addressed more carefully when examining and managing helminth infections in the absence of some critical data on parasite dynamics.

## Author summary

Host-parasite interactions frequently lead to complex dynamics of infection that can be difficult to explain when parasite data are not accurate or complete, which is often the case in natural systems. We used the helminth-rabbit study case and developed a state-space mathematical model to capture the variation in intensity of infection and egg shedding in an experimental trial where hosts were infected weekly, then treated with an anthelminthic and subsequently re-challenged following the same infection regime. Simulations indicate that hosts control the parasite proportionally to the accumulated force of infection and intensities decrease both in the pre- and post-treatment phase. The peak and mean intensity of infection is similar before and after treatment; however, the degree of egg shedding declines proportionately with parasite body length and both traits are lower in the post- than the pre-treatment phase. The intensity of infection fails to adequately explain the variation in the degree of shedding within and between hosts. Instead, this pattern is captured by changes in parasite body length. More attention should be given to the host-parasite interactions within hosts and the impact of external perturbation on the dynamics of infection and transmission.

## Introduction

The large variation in disease severity and transmission often observed among individuals infected with helminths is strongly determined by the local conditions that incoming and established parasites encounter within the host, in addition to variation in the exposure of hosts to infective stages [1–3]. These conditions are mainly determined by the current and previous immuno-physiological attributes of the host, such as the local immune profile or the level of chemical homeostasis, and ecological parasite processes mostly driven by intensity-dependent competition for resources [1, 4–15], where intensity represents the number of parasites in infected hosts. Host and parasite constraints have been shown to affect both the intensity and the life history of parasites with interactions that frequently lead to non-linear dynamics of infection and complex trade-offs between parasite life-history traits.

The within-host regulation of gastrointestinal helminths, and the consequences for the dynamics and life history of the parasite, remains a subject of ongoing research [4, 16–19]. The fundamental challenge is to provide convincing evidence for the mechanisms that affect the intensity of infection and the way parasite traits (i.e. development, fecundity and shedding) adjust to these changes. Ultimately, the understanding of these processes under ecological and immunological forces, and their relative contribution to the observed phenotype of infection, is important for explaining the often large variation in the host’s ability to control the infection.

A useful approach to disentangling the contribution of these two forces is by examining how parasites adjust to external perturbations, such as anthelminthic treatments. Anthelminthics are commonly delivered over one to several days (depending on the type of treatment) and parasites are removed within hours or multiple days; however, in endemic areas reinfection is inevitably the norm. Because of this, by altering the number of parasites competing for host resources and/or the strength of intensity-dependent immune responses [20, 21] treatments lead to patterns of infection and re-infection that can be fundamentally different [2, 4, 22] than treatment-free settings. Indeed, the transient disruption caused by the drug treatment resets the background environment by placing the initial parasite population to 0, thus upon re-infection the new incoming parasites face a primed environment where competition for resources is minimal and/or the immunity still holds some memory from the previous history of infection. Any change in the dynamics of parasite establishment from the pre-treatment to the post-treatment phase should be due to the additional effects of memory in the immune response. In contrast, no changes in parasite dynamics between treatments should be indicative of a regulation by ecological forces driven by intensity-dependent processes in the parasite population.

Irrespective of the anthelminthic treatment, following the constant exposure to infective stages and the accumulation of parasites within the host, the intensity of infection will exhibit a logistic growth with host age if regulated by intensity-dependent ecological forces, such as processes of competition for space and food [4]. A hump-shaped age-intensity profile, where older hosts carry fewer parasites, is indicative of an immune response that regulates the parasite population with a strength that increases proportionately with the force of infection [1, 20]. Other processes can generate this convex profile [2, 22] but here we address this shape as an immunity-generated process. Our previous observations in the *Trichostrongylus retortaeformis*—rabbit system have conformed to the latter pattern, indicating immune regulation [12, 23–26].

The interpretation of the relative contribution of these two forces—ecological limitation through parasite competition for resources and host immune regulation—have conventionally relied on either cross-sectional measures of parasite intensity or longitudinal measures of egg shedding. However, both of these approaches have limitations. For instance, the common method of using age-related, cross-sectional measures of parasite intensity, from dead or drug-treated hosts, assumes that sequential cross-sectional observations in different animals approximate the temporal progression of parasite intensity in a single or average animal and is indicative for a time series of infection [1, 10, 21, 22, 27–31]. This necessarily averages out the within-host heterogeneity in parasite dynamics and traits, as it assumes that the unobservable or hidden dynamics of infection in individuals measured at later times behave similarly to those measured at earlier times. This problem can be circumvented by using time series of parasite shedding, which allows observations of individual host variation during the course of the infection. However, this method requires the assumption that the amount of eggs or larvae shed by a host into the environment is directly proportional to the actual parasite burden [32, 33] and remains constant over time. Yet, an increasing number of studies have shown that this assumption is rarely confirmed or correct [34–43] and the view that the infection-shedding relationship remains constant over time can lead to wrong conclusions. Indeed, any feedback between parasite intensity and fecundity/shedding, or between-host immunity and parasite fecundity, would violate this assumption.

The apparent limitations in the conventional measures of within-host parasite dynamics reflect two challenges in the inference for dynamical systems: the states of interest are often not directly measurable (e.g. parasite burden prior to sacrifice) and/or they are only indirectly measurable (e.g. egg shedding as an indicator of adult parasite burden). State-space or hidden Markov models provide a statistical framework that links a dynamical model of the progression of unobservable states through time to observable measures [44–47]. This class of statistical models has increasingly been used to study the dynamics of infectious diseases at the host population level, where the true incidence of infection is only partially observable through a subset of cases reported through surveillance [48–50].

To understand the relative contribution of immunity and parasite ecological constraints to patterns of helminth intensity, fecundity and shedding, we developed a within-host state-space model of parasite dynamics using a laboratory experiment and the *Trichostrongylus retortaeformis*(TR)-rabbit system. By linking a dynamic model to an observation model and by combining cross-sectional data on TR intensity, body length and fecundity with longitudinal data on egg shedding we are able to reconstruct the unobservable dynamics of infection within each individual and make inference on the underlying dynamic processes. Specifically, to evaluate changes in TR dynamics and traits following external perturbations and provide a mechanism of regulation we compare the model performance before and after anthelminthic treatment and under constant exposure to infective stages.

## Results

### Observations from cross-sectional and longitudinal data

**Fig 1:**
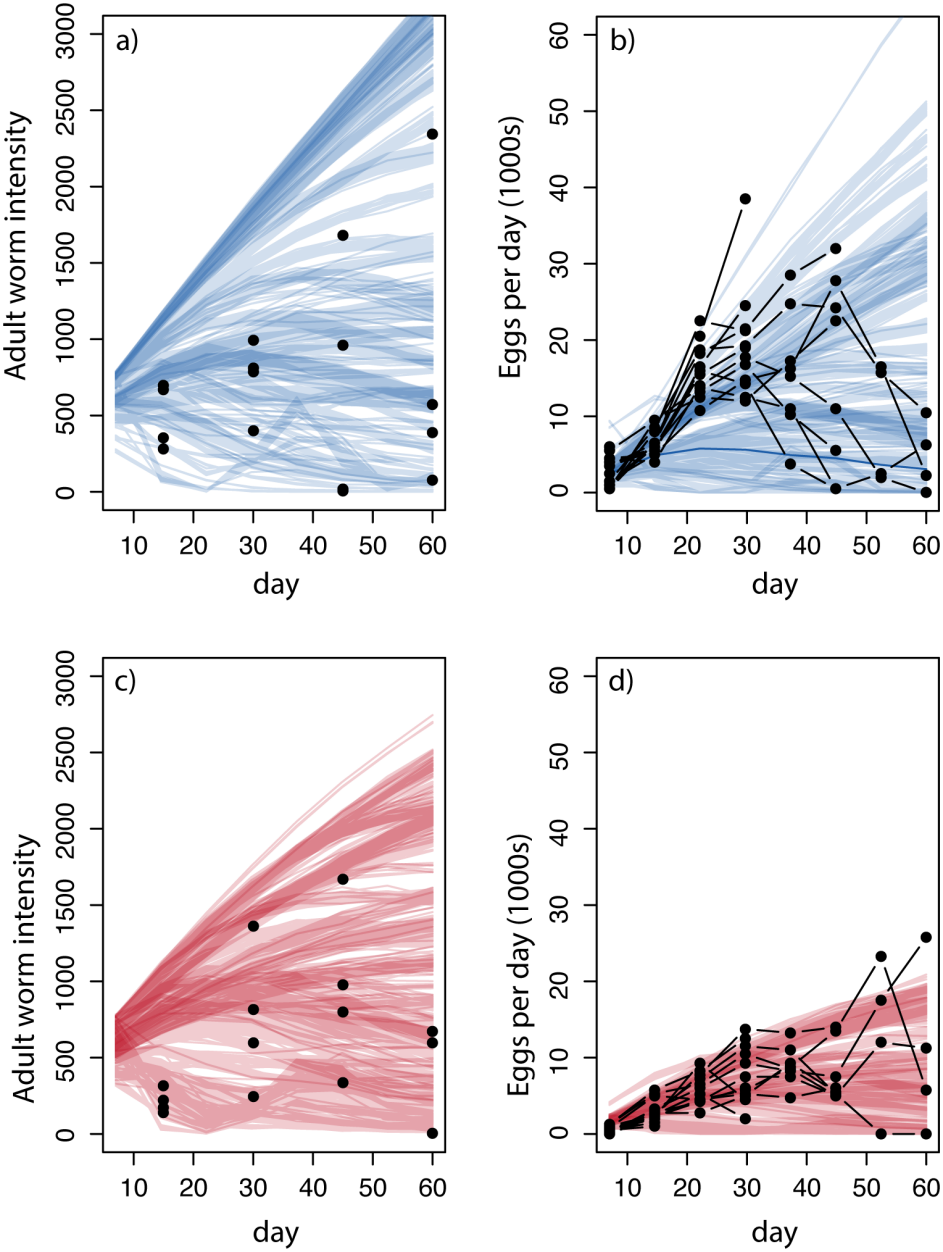
Observed and simulated adult worm intensity and parasite fecundity at each sampling point. (a) Circles give the estimated adult parasite intensity, 4-times the number of worms counted, at each sampling time during the pre-treatment phase. Lines indicate simulated adult parasite intensity each day. Each line reflects a simulation based on a single parameter draw from the posterior density; line shading reflects likelihood weight (darker is higher likelihood); clusters of lines reflect clustering of parameter values for individual animal fits. (b) Circles give the estimated parasite fecundity, EPG divided by 0.03, at each sampling time during the pre-treatment phase. Points are connected indicating repeated observations from a single rabbit. The abrupt truncation of the time series is due to animal sampling. Lines indicate simulated parasite fecundity each day. Shading and clustering of lines are as in (a). (c) Estimated adult parasite intensity for the post-treatment phase. Circles and lines are as in (a). (d) Estimated parasite fecundity for the post-treatment phase.

The regular exposure of rabbits to a constant amount of TR infective larvae leads to a convex pattern between the intensity of infection and host age (i.e. sampling time) both in the pre- and post-treatment phase of the experiment (Fig 1). This is clearer for some rabbits, which is consistent with our previous work [22, 51, 55]. There was no significant difference between pre- and post- treatment in the mean and maximum intensity of infection, controlling for sampling date (ANOVA p-value = 0.528, for additional results see S2 Table). The convex age-intensity profile in both experimental phases, slower parasite accumulation, and delayed increase in egg shedding in the post-treatment phase, is suggestive of an effect of accumulated exposure on the establishment and/or clearance of TR. However, it is difficult to make a definitive conclusion from the data as they reflect averages from rabbits sampled at a single time point and variation in TR intensity among hosts is high.

**Fig 2:**
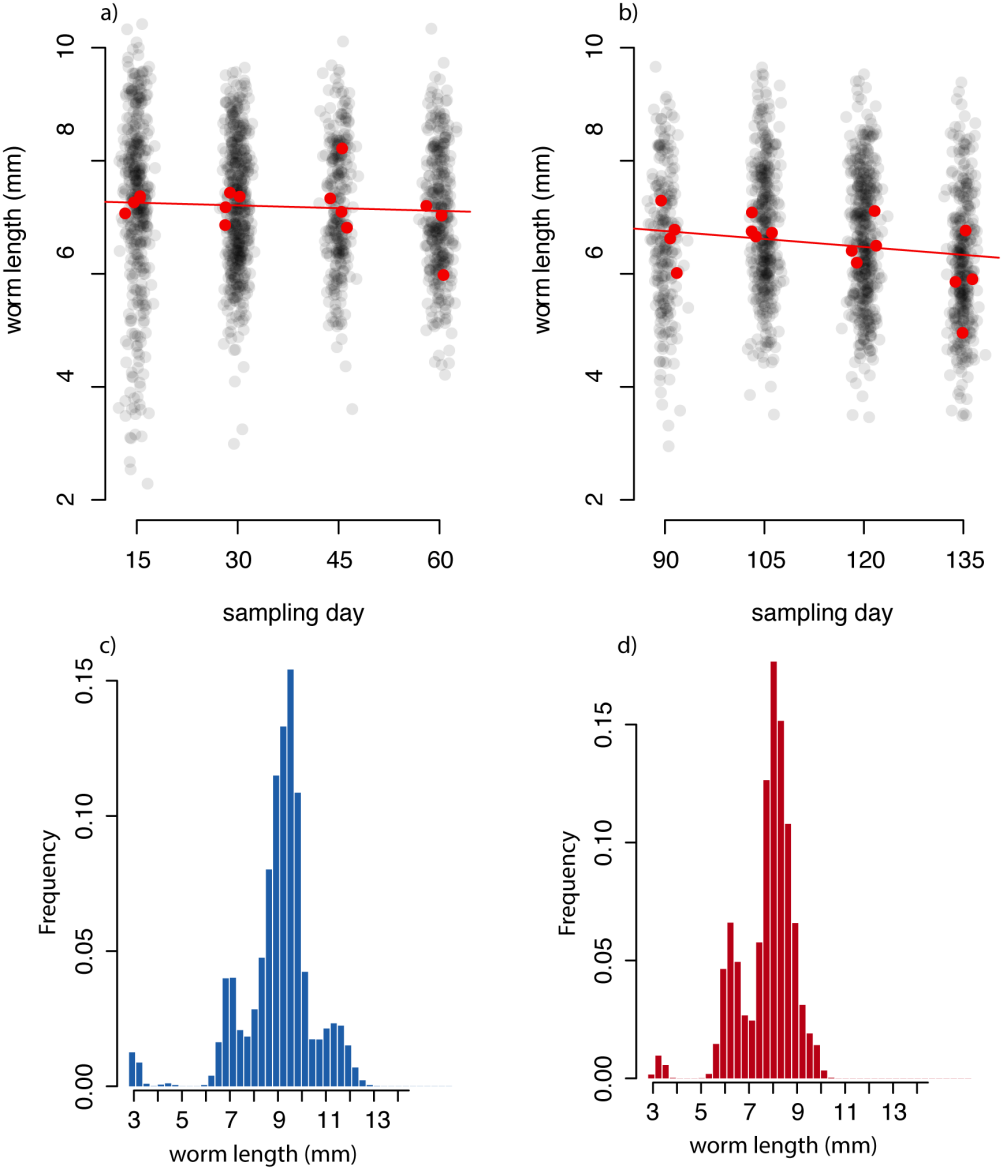
Distribution of observed and simulated worm lengths. (a) The distribution of parasite lengths over time for the pre-treatment phase of the experiment. Black circles give the lengths of all observed worms on each sampling day. Red circles give the mean parasite length for each rabbit. Circles have been jittered in the x-dimension for clarity. The red line indicate the linear trend in the mean parasite length. (b) The distribution of parasite lengths over time for the post-treatment phase of the experiment. Circles and line are as in panel (a)). (c) and (d) The simulated distribution of parasite lengths at day 60 for pre- and post-treatment phases, respectively.

The time series of eggs shed in the feces (EPG) by every animal also exhibited a tendency to a convex pattern with host age (Fig 2(a) and 2(b)). The peak in EPG was reached in approximately 4 weeks in both phases of the infection. The number of eggs shed was significantly lower in the post-treatment (pre- and post-phase, mean = 1842 eggs (range 1000–3850) and 890 eggs (range 475–1325), respectively; mixed effects ANOVA p-value <0.001 with rabbit included as a random effect, S3 Table).

The ratio of the number of eggs in utero per female length was not significantly different between phases (*p* = 0.35; Poisson regression, S2 Fig, S4 Table). However, we found that adult TR were significantly shorter in the post-treatment compared to the pre-treatment phase (body length post- vs pre-phase 95% CI: 6.49 − 6.61 mm vs 7.12 − 7.25 mm Fig 2), after accounting for the effect of sampling time (DPI), the section of the intestine and their interactions (*p* = 0.006; mixed effects ANOVA with rabbit as a random effect; S5 Table); consistent with the overall lower rate of egg shedding in the post-treatment phase.

### Projected effect from the estimated model parameters

**Fig 3:**
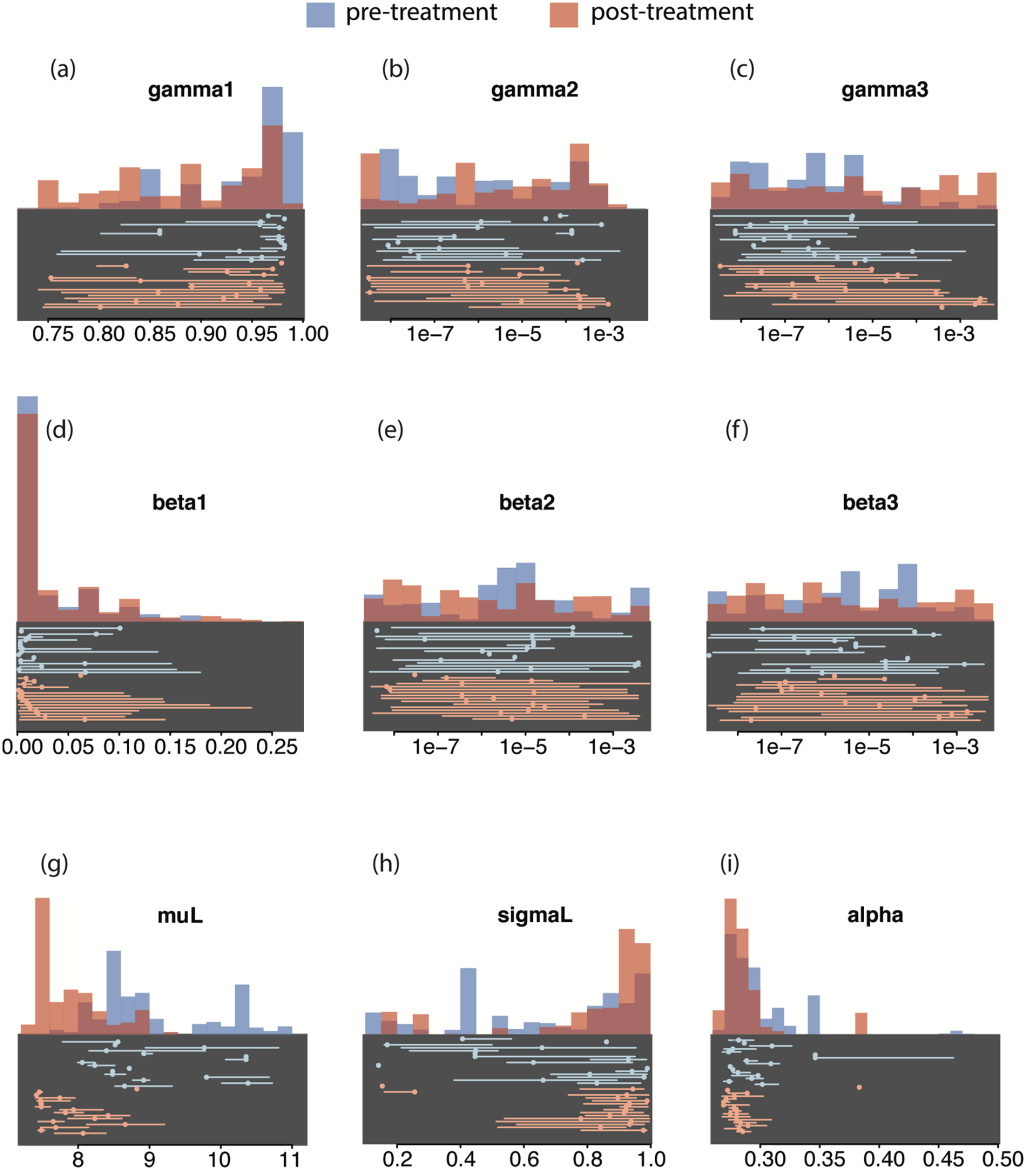
Marginal posterior distributions for parameter estimates. Panels give the sampled posterior distribution, conditional on the observations from all rabbits, for (a) baseline establishment, *γ*_1_, (b) effect of cumulative exposure on establishment, *γ*_2_, (c) effect of parasite intensity on establishment, *γ*_3_, (d) maximum daily clearance, *β*_1_, (e) effect of cumulative exposure on establishment, *β*_2_, (f) effect of parasite intensity on establishment, *β*_3_, (g) mean final length, *μ*_*L*_, (h) variance in final parasite length, and *σ*_*L*_ (i) parasite growth rate, *α*. The posterior for pre-treatment animals is shown in blue, the posterior for post-treatment animals is shown in red. The x-axis in each panel spans the range of the uniform prior for each parameter. Below each panel is shown the posterior mean (circle) for each individual animal and the central 95% of the rabbit-specific posterior distribution. Estimates for animals sampled on days 15, 30, 45, 60 are plotted from top to bottom; pre-treatment animals are shown in blue, post-treatment animals are shown in red.

Forward simulations from the fitted model (Fig 3) replicated the qualitative patterns described by the experimental data. The simulated intensity of infection was comparable between pre- and post-treatment, albeit more variable in the pre-treatment. The number of TR eggs shed was also lower and less variable in the post-treatment, consistent with the original data. Additionally, the fitted model also predicted smaller parasites at 60 days following initial exposure in the post-treatment phase of the experiment, consistent with the observed data (Fig 3(c), see further discussion below).

A total of nine parameters were fitted from the parasite population dynamic and the individual growth models for every rabbit, both in the pre- and post-treatment phase of the experiment. In the population dynamic model, we estimated parameters corresponding to the baseline establishment of infective larval stages (L3), *γ*_1_, adult parasite clearance, *β*_1_, and the degree of either the intensity of adult infection (*γ*_3_, *β*_3_) or the cumulative exposure (*γ*_2_, *β*_2_) on establishment and clearance rates (Table 1). Parameter estimates based on the joint likelihood for the longitudinal and cross-sectional data (Fig 3) indicate that the baseline rate of establishment was reduced in the post-, compared to the pre-treatment phase of the experiment; the posterior mean and central 95^*th*^ quantiles of the fraction of L3 larvae that establish are 0.94 (0.84, 0.98) and 0.88 (0.75, 0.95), respectively (Fig 3—panel (a)). The lower establishment rate in the post-treatment phase is consistent with the later peak in the intensity of TR infection observed. Estimates of adult parasite clearance were low in both the pre- and post- treatment phases of the experiment and the baseline clearance rate was similar in both phases. Overall, parameter estimates were highly variable among rabbits (see points below Fig 3 panels (b), (c), (e), (f)). We note that the posterior mean for larval establishment tended to be lower (Fig 3—panel (a)) and adult clearance tended to have higher mean (Fig 3—panel (c)) in animals sampled later in the experiment. There is a weak trend for a stronger effect of TR intensity and cumulative exposure on establishment (Fig 3—panels (b), (c)) in the animals sampled later in the experiment.

**Table 1:** Mean, 2.5th and 97.5th quantiles of the posterior distribution of model parameters (full-model) in the pre-treatment and post-treatment phase. Note that *γ*_2_, *γ*_3_, *β*_2_, *β*_3_ are presented on a log scale as values are small.

	Pre-treatment			Post-treatment		
	Mean	2.5th	97.5th	Mean	2.5th	97.5th
*γ*_1_	0.94	0.84	0.98	0.89	0.75	0.98
*ln*(*γ*_2_)	-4.1	-8.0	-3.2	-3.9	-8.5	-3.1
*ln*(*γ*_3_)	-4.1	-8.1	-3.2	-3.2	-8.4	-2.5
*β*_1_	0.03	0.00	0.12	0.03	0.00	0.14
*ln*(*β*_2_)	-3.4	-8.4	-2.5	-3.5	-8.3	-2.7
*ln*(*β*_3_)	-3.8	-8.6	-2.9	-3.4	-8.3	-2.5
*μ*_*L*_	9.07	8.06	10.48	7.87	7.42	8.83
*σ*_*L*_	0.66	0.14	0.99	0.81	0.15	0.99
*α*	0.30	0.27	0.35	0.29	0.27	0.38

Averaged over all rabbits in the experiment there was no consistent effect of either cumulative exposure to infective stages or current adult intensity on either larval establishment or adult clearance rate; the posterior distribution is similar to the uniform prior distribution for these four parameters when the model is fit to all rabbit simultaneously. Averaged over all rabbits, there was a strong effect of the treatment on the distribution of parasite lengths; the posterior mean and central 95^*th*^ quantiles of parasite mean final length in the pre- and post-treatment phases are 9.1 mm (8.0, 10.4) and 7.9 mm (7.4, 8.8), respectively (prior mean and 95th percentiles 9.0 mm (7.1, 10.9)).

### Sensitivity analysis

In both sub-models (cumulative exposure submodel and current intensity submodel (S1 Appendix)), the general patterns remained consistent; specifically the larval establishment rate remained lower and mean final body length of parasites were shorter in the post-treatment phase. As in the full model, the posterior mean for the model fit to all rabbits simultaneously showed no strong evidence of an effect of cumulative exposure to infective stages (cumulative exposure sub-model) or TR intensity (current intensity sub-model) on establishment or clearance (S5 Fig).

Compared to the full model with the cross-sectional and longitudinal data, the model fit with longitudinal data only, results in broadly similar patterns in the parameter estimates (S3 Fig). However, the estimated effect of the treatment on establishment of L3 larvae and the estimated adult parasite final length was greater than that from the fit based on the full likelihood for the longitudinal and cross-sectional data.

We can directly compare the observed adult parasite length distribution to the predicted size distribution of parasites at the end of the pre- and post-treatment phases of the experiment for the models fit only to the longitudinal observations (which include no explicit length data) and to both longitudinal and cross-sectional observations. The mean (and IQR) of observed TR lengths at 60 days in the pre- and post- phases was 7.1 mm (6.2, 7.9) and 6.17 mm (5.3, 6.9) respectively; a mean difference of 0.93 mm. For the model fit to both the longitudinal and cross sectional observations, the corresponding predicted mean (and IQR) lengths of adult parasites at 60 days in the pre- and post- phases were 8.1 mm (7.5, 8.7) and 7.0 mm (6.7, 7.5), respectively; a predicted mean difference of 1.1 mm. For the model fit to only the longitudinal EPG observations the corresponding mean (and IQR) lengths of adult parasites at 60 days in the pre- and post- phases were 8.8 mm (8.0, 10.2) and 7.3 mm (7.0, 7.7) respectively; a predicted mean difference of 1.5 mm. Thus, the model fit to only the EPG data, including no explicit length data, estimates a larger effect of anthelminthic treatment on adult parasite length than the full model that includes length data.

### Within-host trends in parasite length

**Fig 4:**
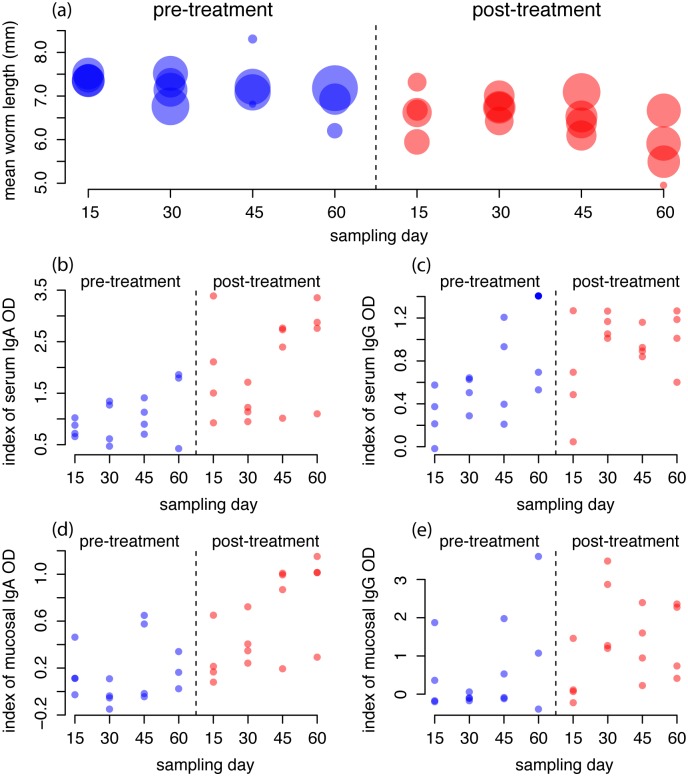
Mean parasite length and antibody levels over time. (a) The mean parasite length per animals at each sampling point post initial infection for the pre-treatment (left) and post-treatment (right) phases. The size of points are scaled by the square root of the number of parasites collected per animal variation in the number of worms in each rabbit. (b) The levels of serum IgA (index of optical density, see Methods) collected on the last sampling point for each animal, (c) the levels of serum IgG collected on the last sampling point for each animal, (d) the levels of mucus IgA collected on the last sampling point for each animal, (e) the levels of mucus IgG collected on the last sampling point for each animal.

Both the empirical analyses and model fits suggest a cumulative effect of time, which here reflects the cumulative exposure to both larvae and adult TR, on TR growth, namely body length. Based on the observed data, we found a significant fixed effect of both treatment and sample day on parasite length in a mixed model with a random effect for rabbit; AIC for the null model of no fixed effects, model with treatment only, and model with treatment and sample day is 9450.1, 9437.5, 9433.7, respectively (Fig 4(a). To investigate the role of host immunity on these patterns, we repeated the same analysis, including the additive effect of IgA and IgG. There was no significant effect of the Igs, suggesting that individual rabbit-level variation in antibody response could not explain variation over and above the cumulative effect over time. We note that when the antibody variables were included in models that did not include sampling day, all were significant; thus time was confounded with the increasing trend in antibody measures across all rabbits, but the host-to-host variation in antibody response did not provide additional explanatory power. We note that the level of both serum and mucosal IgA and IgG increased monotonically over time (Fig 4(b)–4(e)) as mean TR length decreased. We note further that both serum and mucosal IgA and IgG levels at the first sampling date (day 15) of the post-treatment phase were similar to those on the last sampling day (day 60) of the pre-treatment phase (Fig 4(c) and 4(e)), indicating that immunity remained relatively high during the month in which rabbits were not infected.

## Discussion

Experimental infections of rabbits with the intestinal helminth TR showed that animals shed significantly fewer TR eggs following re-infection after anthelminthic treatment. This reduced shedding could arise from fewer established parasites or less fecund females. We showed that the mean number of adult TR was not significantly different between pre- and post- treatment phases. We also showed that the fecundity per female body length relationship was comparable but there was a significant reduction in adult TR body length in the post-treatment phase. We interpret the reduction in observed egg shedding as primarily driven by limitation of TR growth rather than a decrease in parasite number.

However, this descriptive analysis can only provide a limited understanding of the processes generating the experimental patterns and the way they impact the life-history traits of TR. Here, we integrated both cross-sectional and longitudinal data sources in a hierarchical model, which provides a clearer understanding of the within-host constraints on parasite intensity of infection, growth and fecundity. Our modeling results complement our previous empirical findings on this system [12, 51, 54] by showing that the dynamics of TR were likely driven by a limitation in the establishment of infective larvae.

Full model simulations well described the observed data and suggested that parasite establishment was mainly affected by exposure to infective stages, and parasite growth critically affected egg shedding. The convex relationship between host age (i.e. time post infection) and TR intensity, and the significant reduction of body length and eggs shed in the post-treatment phase, are indicative of the immune mediated control of TR, which support the stronger contribution of this driver compared to ecological, parasite intensity-dependent forces, in agreement with our previous work on this system [12, 22, 25, 29, 52]. The IgA and IgG antibody response at the start of the post-treatment phase of the experiment was consistent with that at the end of the pre-treatment phase, suggesting that IgA and IgG decrease relatively slowly following parasite removal and at reinfection parasites experience an already activated immune habitat.

The slower TR establishment and shorter lengths in the post-treatment phase are consistent with limitation by this stronger immune response. We suggest that acquired immunity, which developed proportionally to the accumulated exposure to infective stages over each of the two phases of the experiment, maintained good memory in the absence of infection (i.e. one month) and quickly reacted to the re-infection in the post-treatment phase. This is consistent with the observation that both serum and mucus IgA and IgG levels at the start of the post-treatment phase were at the same level as at the end of the pre-treatment phase. The slower population growth in the post-treatment phase did not facilitate parasite growth and fecundity as would be expected under a model of intensity-dependent limitation; on the contrary, body length was significantly shorter indicating that immunity also affects parasite traits. Moreover, in both phases of the experiment, adult TR clearance tended to be low, consistent with the notion that TR can cope with the hostile local immune environment [26, 62] once established and developed into adults.

We note that rabbit to rabbit variation was high. While there were no consistent effects of TR intensity or cumulative exposure on establishment or clearance across all rabbits, the posterior parameter estimates were highly variable among individual animals. We were unable to identify a strong immune correlate of this individual level variation. In particular, though there was a significant individual level effect of antibody (IgA or IgG, in mucus or serum) on TR length, this effect was not significant when sampling date was included in the model, suggesting that the antibody effect is blurred by sampling time. This lack of a clear relationship should not be interpreted as an absence of immune constraints on TR dynamics and traits. We note that the dynamic nature of the within-host dynamics, the individual variability in immune measures, and the sample sizes used in this experiment may mean this study was under-powered to directly observe an effect of antibody levels on within-host parasite population sizes or life-history characteristics over and above the temporal trends presented here.

Our findings are consistent with previous work on sheep where immunity is likely to delay the maturation of infective larvae resulting in smaller adults at a later time [51]. The immunological basis for the reduced parasite length following re-infection is well described in sheep [55, 63, 64]. A reduction in the number of eggs shed by stunted parasites has been previously reported from field and laboratory work [8, 11, 37, 54, 55, 59, 65–68]. The mechanism commonly proposed in all these studies is a limitation of parasite body growth with increasing levels of exposure, mediated by host and/or parasite processes. There is also evidence that body length and number of eggs in utero increase below a parasite intensity threshold, but above this value the intensity-traits relationship becomes negative [69]. Our findings support these studies but we also show that, immunological constraints, which scale with accumulated exposure, have a relatively stronger impact on parasite traits than parasite intensity-dependent ecological processes. Moreover we show that changes in parasite traits, rather than intensity, better explains the variation in the degree of shedding before and after anthelminthic treatment. Ultimately, these relationships can provide insight of the trophic processes affecting the evolution of parasite resistance, namely, the parasite traits that are most critically constrained and how they are affected over time; for instance, during multiple re-infections [15].

Our analyses of sub models revealed broadly consistent patterns. Sub-models that included only the effects of cumulative exposure to infective stages or adult parasite intensity estimated low larval establishment and shorter mean adult body lengths in the post-treatment phase, consistent with the patterns seen in the full model. An analysis based only on longitudinal observation of eggs shed in feces, an indirect measure of the within-host dynamics that includes no explicit information about parasite body length, gave qualitatively consistent insights about the effect of immune limitation on larval establishment and TR growth, but tended to over-estimate quantitative effects relative to the full model including the direct, cross-sectional observations. This is notable because it suggests that observations of egg shedding alone can inform the general trend in worm intensity through time, but is not sufficient to recover the observed changes in worm traits.

Overall, the population dynamics of *T. retortaeformis* appear to be characterized by a high variation among rabbits, suggesting that caution must be taken in the interpretation of cross-sectional observations. Heterogeneities in the intensity of infection and degree of shedding among hosts have important implications for the accumulation of infective stages in the environment and transmission. Recognizing the mechanisms responsible for such a pattern and, more critically, the components of the parasite life cycle that are more strongly affected, is fundamental for a better understanding of the dynamics of helminth infections and for developing more effective control measures where parasites are endemic. Consistent with previous work [70, 71] we show that, following anthelminthic treatment, parasites regain intensities similar to pre-treatment conditions, albeit more slowly, but parasite fitness is consistently altered. By appreciating that parasite traits do change in response to cumulative exposure, and limitations on parasite traits may persist following external perturbations, it is possible to provide more accurate estimate of the risk of infection. For helminths regulated by host immunity we should expect a decrease in the fitness of the parasite population with host age, even if parasite intensity is reduced through anthelminthic treatment, and overall lower risk of infection irrespective of their parasite load. This trend in parasite fecundity over time, likely in response to the immune response, may have long term effects on parasite persistence and development of drug resistance. This observed pattern would predict that there is a cost to the parasites that develop resistance to drugs, as they would be expected to be shorter and less fecund over time.

### Conclusion

Our modeling framework provides a novel approach to the study of helminth infections at the host level. Indeed, while offering a parsimonious explanation of the forces driving the dynamics of the parasite it also identifies the traits that are primarily constrained and the processes that generate such patterns. Moreover, the impact of external perturbations, namely anthelminthic drug treatment, is well captured in the simulated dynamics of infection. Ultimately, although this modeling approach needs information both on longitudinal and cross-sectional data it can provide quantitative predictions on changes in helminth intensity and traits under testable external disturbance.

## Methods

### Ethics statement

All the animal procedures were approved by the Institutional Animal Care and Use Committee of The Pennsylvania State University (USA).

### The system and the empirical data

The intestinal helminth *Trichostrongylus retortaeformis* (TR) is a common parasite of the European rabbit (*Oryctolagus cuniculus*). Hosts become infected by ingesting herbage contaminated with infective third stage larvae (L3). Larvae colonize the small intestine where they mature into adults that reproduce and shed eggs into the environment through the host’s feces. We previously showed that rabbits develop a strong type 1 and type 2 immune response that can clear or reduce the parasite load, although there is no life-long protection and animals are constantly re—infected under endemic exposure [12, 51–53]. The initial type 1 response is probably a reaction to the bacterial infiltration in the intestinal wall damaged by the movements of larvae across the tissue during maturation [25, 54]. This inflammatory response does not seem to affect the long—term ability of the rabbit to control the infection, which occurs through a type 2 immune response [12, 25]. We also showed that there is a negative relationship between body length, or fecundity (i.e. number of eggs in utero per body length), and both antibody IgA levels and intensity of infection [51, 54]. While immunity plays an important role to the dynamics of this helminth [12, 29, 55], it is still unclear if ecological density-dependent parasite processes also have any impact on parasite dynamics and traits and how they adjust to these host and parasite driven forces under anti-helminthic perturbation.

Here we performed a laboratory experiment where we quantified parasite variables (counts, body length and egg shedding through feces) and immune response (IgA and IgG antibodies) before and after anthelmintic treatment and over a six-month period. Out-bred, New Zealand, 2 months old, male rabbits were housed in single cages with a 12h light cycle and a daily access to 125 g of standard rabbit pellets and water *ad libitum*. Animals (n = 36) were orally trickle dosed every 7 days with 400 infective third stage larvae (L3) suspended in 5 ml of tap water; control animals (n = 12) only received tap water. In the current study we focus only on the infected rabbits; the feces of control animals were routinely monitored for TR eggs and were found to be zero at all time points and no parasites were found in the intestine of control animals at sacrifice; we take this as evidence of the independence of infections in rabbits. Groups of four infected animals were sacrificed at days 15, 30, 45 and 60 post initial infection. At day 60, animals were orally treated for five consecutive days with the broad spectrum anthelminthic Fenbendazole at dosage adjusted by animal body mass (5mg fenbendazole/kg body weight, based on a 10% suspension (100 mg/ml)) (Panacur, Intervet Inc., USA). Infection was then suspended for 30 days, including the 5 day drug treatment, and subsequently reinstated following the same infection procedure and sampling frequency with four animals sacrificed at days 90, 105, 120, 135 and 150 post initial infection. The removal of TR by the anthelminthic drug means that L3 larvae introduced in the post-treatment phase experience the same population dynamic conditions as in the pre-treatment, though may experience a novel immunological environment due to the history of prior exposure. We selected a one-month hiatus in the re-infection to allow the immune response enough time to decrease in the absence of parasites and mimic the natural condition of lack of exposure for a limited period, but one that was longer than the weekly dosing. This experimental design allows us to investigate the relative contribution of coupling between ecological and immunological forces within the host. Specifically, it provides a context for the effect of host immunity (which necessarily depends on parasite intensity) as well as parasite population on parasite establishment, development and reproduction of new parasites in nature. The treatment successfully removed all the parasites as no eggs were found in the feces during the one month following anthelminthic treatment also no parasites were found in the intestine of rabbits sampled prior to the start of the post-treatment phase at day 0 (or 90 days post initial infection). The sampling points were selected to be compatible with the helminth life cycle (pre-patent period about 12 days) and to provide information on the parasite variables over the course of the infection. At every sampling point, animals were processed as described previously [25]. Briefly, the small intestine was divided into four sections (from duodenum to ileum: SI–1 to SI–4) and the parasites from one half of each section were collected in a 50mL tube and then counted and sexed in 10x2.5 ml aliquots from that tube (i.e. raw counts reflect 50% of worms in 50% of each section of the intestine). For every section, a sample of randomly selected specimens (≈ 50 parasites for each sex) was stored in 10% PBS—buffered formalin and subsequently processed for biometry data (body length (male and female) and number of eggs in female’s uterus) [51, 54]. Along with these cross—sectional data, we also collected longitudinal records on the number of parasite eggs shed in feces of every host twice a week starting from the second week post initial (re–) infection, following standard parasitological procedures (eggs per gram of feces, EPG, [56]). In summary, for each host we collected four distinct parasite measurements: total count from a fraction of intestine, body length (male and female) and eggs in (female’s) uterus at the time of individual sampling, as well as eggs shed through host’s feces every week prior to rabbit sampling.

Parasite-specific antibody responses (IgA and IgG) were measured in the blood serum (every week) and in the duodenum mucus (at sampling points) using indirect ELISAs. We used homogenates of adult TR as the source of antigen, as described previously [25, 51]. The following dilutions were used: IgA mucus extracts 1: 10, serum samples 1: 20; IgG mucus 1: 20 and sera 1: 160. Briefly, all dilutions were prepared in 1% (w/v) non-fat milk diluted in Phosphate buffered saline (ph7.2) supplemented with 0.05% Tween-20 (Fisher Scientific, Hampton, NH). Antigen-antibody complexes were allowed to form overnight at 4°C prior to addition of anti-rabbit IgA or IgG detection antibody (diluted 1:2000 in the same buffer that was used for diluting test samples). Quantification was based on spectrophotometric analysis; antibody values were then transformed and standardized into optical density (OD) indexes based on positive and negative control samples that were included in each assay plate, see full details reported in our previous work [25, 51, 57]. For simplicty we refer to the OD index as “antibody levels”.

### The model framework

To examine the model for the within-host dynamics of infection and development of TR, we applied a state-space modelling framework that linked the quantified data of parasite intensity—a direct but cross-sectional measure of the state of interest, and time series of fecal egg counts—an indirect longitudinal measure of parasite intensity—to dynamic models that describe the time progression of the unobservable states (parasite counts and size) via an observation model [58].

### Dynamic models

We modeled the dynamics of parasite intensity within the host as a birth-death process (i.e. establishment of infective L3 larvae and parasite mortality/clearance), where the rates of establishment and clearance are assumed to depend on both cumulative exposure to L3 larvae and current adult parasite intensity. The population of adult parasites at time *t* + 1, *P*_*t*+1_ is the result of the combined effect of cumulative exposure and parasite clearance

$$P_{{}_{t+1}}=\Lambda \gamma_{{}_{1}}\text{exp}\left(-\gamma_{{}_{2}}\sum_{t}\Lambda -\gamma_{{}_{3}}P_{{}_{t}}\right)+P_{{}_{t}}-\beta_{{}_{1}}\left(1-\text{exp}\left(-\beta_{{}_{2}}\sum_{t}\Lambda -\beta_{{}_{3}}P_{{}_{t}}\right)\right)P_{{}_{t}}$$ (1)

where Λ is the known force of infection (i.e. weekly L3 dose: assumed to be 0 on the days when larvae are not administered and 400 on the days of infection), *γ*_1_ is the baseline probability of parasite establishment, *γ*_2_ and *γ*_3_ give the per capita reduction in L3 establishment due to either new L3 larvae or established adults, respectively. The baseline probability of clearance is given by *β*_1_, *β*_2_ and *β*_3_ give the per capita increase in clearance rate (e.g. adult mortality) due to new L3 larvae or established adults, respectively.

The expected body length (in mm) of individual parasites at time t, *x*_*t*_ is described by the discrete time logistic model [59],

$$x_{{}_{t+1}}=x_{{}_{t}}+\alpha x_{{}_{t}}\left(1-x_{{}_{t}}/L\right)$$ (2)

where *α* is the mean developmental rate in length and *L* is the mean final length of an adult parasite. The distribution of parasite length at age *t*, is assumed to be normal with expectation *μ*_*L*_ and variance $\sigma_{{}_{L}}^{2}$. Based on our lab measurements we assumed that all L3 larvae start at an initial mean length *x*_0_ of 1.069 mm and standard deviation of 0.062 (a total of 53 L3 larvae were sampled to estimate the length distribution at the start of the experiment). The starting L3 size distribution is assumed to be constant at all times during the experiment. Adult male parasites are assumed to have mean final length that is 0.75 times that of adult female parasites which is ≈ 8*mm* [51, 54]; the mean final length *μ*_*L*_ reflects the average of all male and female parasites assuming a 1:1 sex ratio.

### Observation models

For every rabbit, and at each sampling point, parasite counts from one quarter of each of the 4 small intestine sections were combined and the observed number of parasites at time *t*, *C*_*t*_, was thus assumed to be distributed as follows:

$$C_{t}∼\text{Binomial}\left(P_{{}_{t}},0.25\right)$$

where *P*_*t*_ is the observed parasite intensity in the whole small intestine at the time of sampling in that specific rabbit. The observed counts of parasites at each length, was binned into length classes of 0.25 mm intervals between 2 and 20 mm. The resulting vector *Z*, of length 72, with elements *z*_*l*_ is assumed to be drawn from a multinomial distribution with probability vector equal to the proportion of simulated parasites in each size class. Binning the lengths into these discrete classes allows the use of a multinomial likelihood, with expectation determined by the simulated length distribution, without assuming an explicit functional form of distribution of parasite lengths.

The number of parasite eggs shed by every host (EPG, based on the average of 2 measurements a week) is a partial observation of the total number of parasite eggs, *E*_*t*_ discharged by an animal on a given day. EPG counts are assumed to reflect approximately 3% of the average daily fecal production by a rabbit (based on 30g of dry feces from laboratory rabbits of similar age and under the same feeding regime, unpublished data) and assumed to be uniform across the different animals and constant over the day (see S4 Fig for sensitivity of fitted parameters to the assumed observation rate). The observed number of eggs shed is assumed to be distributed as:

$${\text{EPG}}_{t}∼\text{Poisson}\left(0.03E_{t}\right).$$

This makes the implicit assumption that the rate of eggs shed is homogeneous through time and that the sampled feces are representative of all feces from a given animal.

Parasite fecundity (i.e. the number of eggs in utero per adult female parasite at sampling day *t*) is known to be positively correlated with parasite length [51, 54, 60]. The observed relationship between eggs in utero and female parasite length(S2 Fig, S4 Table) was fit using a generalized linear mixed model (GLMM), with Poisson error distribution. We also tested whether the slope of this relationship changed from the pre-treatment to post-treatment phase of the experiment. In the simulation model, the total number of eggs produced by every female at time *t* was a random draw from the fitted GLMM, conditional on the predicted length of that female. Only body lengths longer than 4mm, the length at which females are assumed to be sexually mature, were considered (no eggs were observed in females smaller than 4mm throughout the experiment (S1 Fig)). The estimated fecundity of the total helminth population at time *t*, *E*_*t*_ was then quantified as the sum of eggs over all adult females, predicted by the population dynamic (Eq (1)) and individual growth (Eq (2)) models.

### Model fitting

We used a Bayesian particle filter method [61] to estimate the parameters of the parasite population dynamic model (Eq (1)) and the individual parasite growth model (Eq (2)) independently for each rabbit. The posterior distribution for all parameters was estimated by sampling from a large (50, 000) set of parameter combinations with probability proportional to the product of the joint prior probability and the approximate likelihood, where the latter is calculated via a particle filter (explained below), for each parameter combination. Candidate parameters were sampled from conditionally independent uniform distributions. The upper and lower bounds on the uniform prior distributions were chosen to limit dynamics to the range of observed data.

We estimated an approximate likelihood for each parameter combination using a particle filter [49]; an algorithm for estimating the likelihood for models where the exact likelihood cannot be stated analytically but realizations of the model can be generated by simulation [50]. These methods have a long history in signal processing and have been recently applied in a number of population dynamic and epidemiological scenarios with partial or imperfect observation of state variables [44–47]. The evaluation of the estimated likelihood requires two linked models: a dynamic model to generate the forward projection of the state variables—here the within-host parasite intensity and body length distribution (Eqs (1) and (2))—and an observation model to evaluate the likelihood of observing the data—in our case—parasite counts, EPGs and parasite lengths, conditional on the values of the unobservable state variables.

The steps below outline the approximation of the likelihood for each parameter combination, *ϕ*_*k*_ for *k* = 1…50, 000 for a single rabbit. We treat all rabbits as statistically independent and thus the likelihood of parameter combination given the observations for all rabbits is the product of the individual rabbit likelihoods; below we present estimates conditional on the observations for all rabbits jointly, and each rabbit independently. The evaluation of the particle filter for one parameter combination returns an estimated likelihood, which is used in the calculation of the posterior sampling probability. To evaluate the particle filter for the longitudinal observations of EPG for rabbit *r* conditional on one specific parameter combination the following approach was implemented:

1. We initialize simulations by assuming an initial dose of 400 L3 larvae, with lengths distributed as Gaussian with mean 1.069 mm and standard deviation of 0.062 mm (see above).
2. We simulate N stochastic realisations (termed particles) of the system in daily time steps up to day 7 (i.e., the interval between L3 dose and EPG measurement)—the parasite population are simulated according to Eq (1), and their related body-lengths according to Eq (2).
3. We then generated *N* stochastic realizations of total EPG corresponding to the simulated parasite population sizes and length distributions by taking random draws from the egg-length model (S2 Fig) for the simulated adult female parasite population with length greater than 4 mm (we considered only observed eggs in females longer than 4mm, (S2 Fig)) and summing over all females.
4. We evaluated the likelihood of the observed EPG at that observation step conditional on each simulated particle (i.e. for simulated particle, *i*, the EPG_*t*_ ∼ Poisson(0.03*E*_*t*_)), where *i* varies from 1 to *N*). The mean likelihood over all *N* particles gives an estimate of the likelihood of the EPG observation at time *t*.
5. Finally, we re-sampled the simulated particles (i.e. the simulated trajectories of parasite population size, length distribution and total EPG), with replacement, using sampling probabilities proportional to the likelihood of the observed EPG for each particle.
6. We then return to step (2) and simulate parasite intensity and size distribution forward to the next observation point, taking the re-sampled particles from step (5) as the new starting point.

This process was repeated for all EPG observation points; the average of the likelihood of all *N* particles at each time point gives an estimate of the conditional likelihood of the observed EPG at time step *t*. The product of these conditional likelihoods, for all observations, 1 to *T*_*r*_, (where *T*_*r*_ is the time of sacrifice for rabbit *r* in days) is then the estimated joint likelihood of the longitudinal EPG observations from each rabbit for a single parameter combination $\prod_{t=1}^{T_{r}}L\left(E_{t}|{\text{EPG}}_{t}\right),\phi_{k})$.

On the final observation—the day of animal sacrifice, we evaluated the likelihood of the cross-sectional observations of parasite intensity and parasite length. The conditional likelihood of the observed parasite intensity ($O_{T_{r}}$) was calculated from the average of the likelihood for each *N* simulated parasite intensities ($P_{T_{r}}$), which is given by $L(O_{T_{r}}|P_{T_{r}},\phi_{k})$, assuming $O_{T_{r}}∼\text{Binomial}(P_{T_{r}},0.25$).

The conditional likelihood of the observed parasite lengths ($X_{T_{r}}$) was calculated given the average of the likelihood for each of the *N* simulated lengths distributions ($Z_{T_{r}}$), is given by $L(X_{T_{r}}|Z_{T_{r}},\phi_{k})$, assuming that the observed vector of counts in length classes is a Multinomial draw, with trials equal to the number of observed adult parasites and probability vector equal to the proportion of simulated parasites at the final time step, *T*_*r*_, in each size class for particle *i*. The product of these two likelihoods gives the estimated likelihood of the cross-sectional observations from each individual rabbit for a single parameter combination.

The product of the estimated likelihoods for the longitudinal and cross-sectional observations gives the total likelihood of all observations for a single parameter combination for each rabbit. We assume that all rabbits are independent, thus the total likelihood for parameter combination *ϕ*_*k*_ is the product of the likelihoods for all, *R*, rabbits

$$L^{R}|\phi_{k}=\prod_{r=1}^{R}(\prod_{t=1}^{T_{r}}L\left(E_{t}|EPG_{t},\phi_{k}\right)\left(L\left(O_{T_{r}}|P_{T_{r}},\phi_{k}\right)\right)\left(L\left(X_{T_{r}}|Z_{T_{r}},\phi_{k}\right)\right)).$$

As many parameter combinations have very low likelihood, we implemented a two-step algorithm for computational convenience. We first estimated the likelihood for all parameter combinations using *N* = 100 particles and discarded all parameter combinations with estimated likelihood less than 1% of the maximum. We then re-estimated the likelihood for the remaining parameter combinations using *N* = 1000 particles, to obtain a more precise estimate of *L*^*R*^.

After estimation of the likelihood for each parameter combination, we generate draws from the posterior distribution by re-sampling the parameter combinations, with replacement, using probabilities proportional to the likelihood for all rabbits. We compare this posterior distribution to that derived by re-sampling parameter combinations, with replacement, using probabilities proportional to the likelihood of the longitudinal observations only; e.g. assuming the cross-sectional data were not available.

We also generated rabbit-specific parameter estimates by re-sampling parameter combinations, with replacement, using probabilities proportional to the likelihood for each rabbit independently.

We fitted the same model framework to the pre- and post-treatment phase independently, assuming no explicit effects on the post-treatment dynamics from the pre-treatment conditions; as such any effect of the pre-drug exposure would be reflected in the estimated rates and strength of cumulative exposure on establishment and clearance.

To examine the single effect of either cumulative exposure of infective stages or intensity of adult infection on explaining the dynamics observed, we fitted sub-models that set the effects of cumulative exposure (*γ*_2_ and *β*_2_) or the effects of parasite intensity (*γ*_3_ and *β*_3_) to 0 (S5 Fig).

## Supporting information

S1 Appendix Sub-model frameworks for the parasite population dynamics. Here, we present the two sub-models that are nested within the full population dynamic model presented in the main text.
(PDF)

S1 Fig Schematic of the experimental setup. We performed a laboratory experiment by infecting the European rabbits (*Oryctolagus cuniculus*) with their common intestinal helminth *Trichostrongylus retortaeformis*. Animals were orally trickle dosed every 7 days with 400 infective third stage larvae (L3) where control animals were orally treated with tap water. Groups of 4 infected and 2 control rabbits were sacrificed at days 15, 30, 45 and 60 post initial infection. At day 60, animals were orally treated for 5 consecutive days with the anti-helminthic Fenbendazole at dosage adjusted by body mass. Infection was then suspended for 30 days and subsequently reinstated following the same infection procedure and sampling frequency with 6 animals sacrificed at days 105, 120, 135 and 150 post initial infection. For each individual animal we collected three distinct parasite measures: abundance, body length and eggs in utero at the time of sampling (i.e. cross-sectional data) and eggs shed weekly (longitudinal data) starting from the second week post initial infection prior to sampling. Together with parasite data we also quantified host immune measures—like IgA and IgG (mucus and serum both).
(TIF)

S2 Fig Relationship between eggs in utero and adult parasite body length in females from the pre(solid curve)- and post(dashed curve)-treatment phase. A random number of *T. retortaeformis* specimens were collected from the small intestine of rabbits at every sampling point and their body length and number of eggs in utero -for the females- were measured using our established protocol [51, 54]. Eggs in utero were positively correlated to female body length (Generalized Linear Mixed model, GLMM, with a log-link function, Poisson error distribution and rabbit as a random effect)(S4 Table), however, this relationship was not significantly different in the pre- and post-anthelminthic treatment phases of the experiment (p = 0.35). Grey shading gives a 95% prediction interval.
(TIFF)

S3 Fig Parameter estimates from EPG (the longitudinal data) only. Panels give the sampled posterior distribution, conditional on the observations from all rabbits, for (a) baseline establishment, *γ*_1_, (b) effect of cumulative exposure on establishment, *γ*_2_, (c) effect of parasite intensity on establishment, *γ*_3_, (d) maximum daily clearance, *β*_1_, (e) effect of cumulative exposure on establishment, *β*_2_, (f) effect of parasite intensity on establishment, *β*_3_, (g) mean final length, *μ*_*L*_, (h) variance in final parasite length, and *σ*_*L*_ (i) parasite growth rate, *α*. The posterior for pre-treatment animals is shown in blue, the posterior for post-treatment animals is shown in red. The x-axis in each panel spans the range of the uniform prior for each parameter. Below each panel is shown the posterior mean (circle) for each individual animal and the central 95% of the rabbit-specific posterior distribution. Estimates for animals sampled on days 15, 30, 45, 60 are plotted from top to bottom; pre-treatment animals are shown in blue, post-treatment animals are shown in red.
(TIF)

S4 Fig Sensitivity analysis of the parameters. In the main text, we present a model that assumes 3% of eggs shed in the feces are counted, based on 30g dry weight of feces produced per day. Each panel gives the central 95% of the sampled posterior distribution for pre-treatment (lower line) and post-treatment (upper line) animals assuming that observed egg shedding was 50% lower (low), and 50% higher (high) than the assumption made in the main text (null). Individual panels indicate each parameter (a) baseline establishment, *γ*_1_, (b) effect of cumulative exposure on establishment, *γ*_2_, (c) effect of parasite intensity on establishment, *γ*_3_, (d) maximum daily clearance, *β*_1_, (e) effect of cumulative exposure on clearance, *β*_2_, (f) effect of parasite intensity on clearance, *β*_3_, (g) mean final length, *μ*_*L*_, (h) variance in final parasite length, and *σ*_*L*_ (i) parasite growth rate, *α*. The absence of lines indicates a model for which a given parameter was not present.
(TIF)

S5 Fig Comparison of full and sub-models. In the main text, we present a model with effects of both cumulative parasite burden and parasite intensity on both larval establishment and adult clearance. Each panel gives the central 95% of the sampled posterior distribution for pre-treatment (lower line) and post-treatment (upper line) animals for the intensity-only (int), exposure-only (exp), and full models (full). Individual panels indicate each parameter (a) baseline establishment, *γ*_1_, (b) effect of cumulative exposure on establishment, *γ*_2_, (c) effect of parasite intensity on establishment, *γ*_3_, (d) maximum daily clearance, *β*_1_, (e) effect of cumulative exposure on clearance, *β*_2_, (f) effect of parasite intensity on clearance, *β*_3_, (g) mean final length, *μ*_*L*_, (h) variance in final parasite length, and *σ*_*L*_ (i) parasite growth rate, *α*.
(TIF)

S1 Table Prior distributions for model parameters. Prior distributions were uniform on a transformed scale.
(PDF)

S2 Table ANOVA table to test alternate models for total number of adult parasites per rabbit. The base model includes only the sampling date (DPI), subsequent models include all variables in the rows above.
(PDF)

S3 Table Likelihood ratio tests of alternate models for Egg per Gram (EPG) of feces. The base model includes only the sampling date (week), subsequent models include all variables in the rows above. In all models, the rabbit from which parasites were sampled, was treated as a random effect.
(PDF)

S4 Table Summary table for the GLMM that represents the relation between eggs in the utero and parasite body length in S2 Fig. The number of eggs per female parasite is modeled as a function of parasite length (mm) and experimental phase (pre- and post-) with a random effect the rabbit from which parasites were sampled. We used a log-link function and Poisson error distribution.
(PDF)

S5 Table Likelihood ratio tests of alternate models of female parasite length. The base model includes only the sampling date (DPI), subsequent models include all variables in the rows above. In all models, the rabbit from which parasites were sampled was treated as a random effect.
(PDF)
