# Supplementary material for: Changes in parasite traits, rather than intensity, affect the dynamics of infection under external perturbation
Source: PLoS Comput Biol. 2018 Jun 11;14(6):e1006167. doi: 10.1371/journal.pcbi.1006167 (PMC6019670; doi:10.1371/journal.pcbi.1006167)
Supplement: S1 Appendix — Here, we present the two sub-models that are nested within the full population dynamic model presented in the main text. (PDF) [file pcbi.1006167.s001.pdf]

Changes in parasite traits, rather than intensity, affect the dynamics of infection under external perturbation: Supplementary Information

Suma Ghosh[1], Matthew J. Ferrari[2], Ashutosh K. Pathak[3], and Isabella M. Cattadori[2]

[1]Department of Mathematics, School of Natural Sciences, Shiv Nadar University, UP, INDIA
[2]Center for Infectious Disease Dynamics, The Pennsylvania State University, PA, USA
[3]Odum School of Ecology, The University of Georgia, GA, USA

May 27, 2018

S.1 Supporting text

§1. Partial-model frameworks for the parasite population dynamics

Here, we present the two sub-models that are nested within the full population dynamic model presented in the main text; specifically:

Full Model:

$$P_{t+1} = \Lambda\gamma_1 \exp(-\gamma_2 \sum_t \Lambda - \gamma_3 P_t) + P_t - \beta_1(1 - \exp(-\beta_2 \sum_t \Lambda - \beta_3 P_t))P_t \quad (1)$$

where Λ is the known force of infection (i.e. weekly L3 dose: assumed to be 0 on the days when larvae are not administered and 400 on the days of infection). The parameters are described in the Table S1.

In the sub-models, we assume that host immunity develops either as a function of accumulated exposure to infective stages or current intensity of adult infection. In the first scenario, we assume that parasite establishment and clearance scale only with the accumulated parasite exposure:

Cumulative exposure sub-model:

$$P_{t+1} = \Lambda\gamma_1 \exp(-\gamma_2 \sum_t \Lambda) + P_t - \beta_1(1 - \exp(-\beta_2 \sum_t \Lambda))P_t \quad (2)$$

In the second scenario, we assume that parasite establishment and clearance scale only with the current adult intensity:

Current intensity sub-model: Current intensity of infection as a proxy of host immunity

$$P_{t+1} = \Lambda\gamma_1 \exp(-\gamma_3 P_t) + P_t - \beta_1(1 - \exp(-\beta_3 P_t))P_t \quad (3)$$
