# Supplementary figures and images for: Changes in parasite traits, rather than intensity, affect the dynamics of infection under external perturbation
Source: PLoS Comput Biol. 2018 Jun 11;14(6):e1006167. doi: 10.1371/journal.pcbi.1006167 (PMC6019670; doi:10.1371/journal.pcbi.1006167)

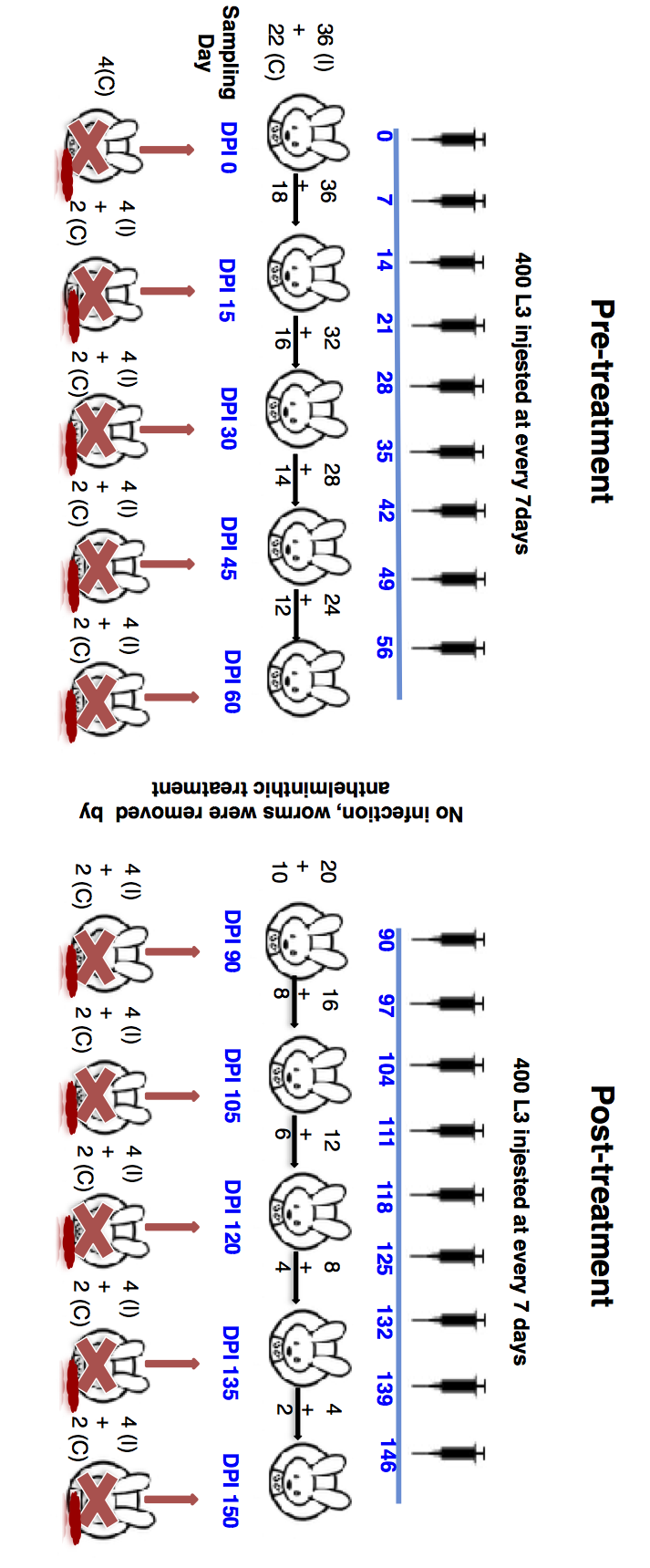

Supplement: S1 Fig — We performed a laboratory experiment by infecting the European rabbits (Oryctolagus cuniculus) with their common intestinal helminth Trichostrongylus retortaeformis. Animals were orally trickle dosed every 7 days with 400 infective third stage larvae (L3) where control animals were orally treated with tap water. Groups of 4 infected and 2 control rabbits were sacrificed at days 15, 30, 45 and 60 post initial infection. At day 60, animals were orally treated for 5 consecutive days with the anti-helminthic Fenbendazole at dosage adjusted by body mass. Infection was then suspended for 30 days and subsequently reinstated following the same infection procedure and sampling frequency with 6 animals sacrificed at days 105, 120, 135 and 150 post initial infection. For each individual animal we collected three distinct parasite measures: abundance, body length and eggs in utero at the time of sampling (i.e. cross-sectional data) and eggs shed weekly (longitudinal data) starting from the second week post initial infection prior to sampling. Together with parasite data we also quantified host immune measures—like IgA and IgG (mucus and serum both). (TIF) [file pcbi.1006167.s002.tif]

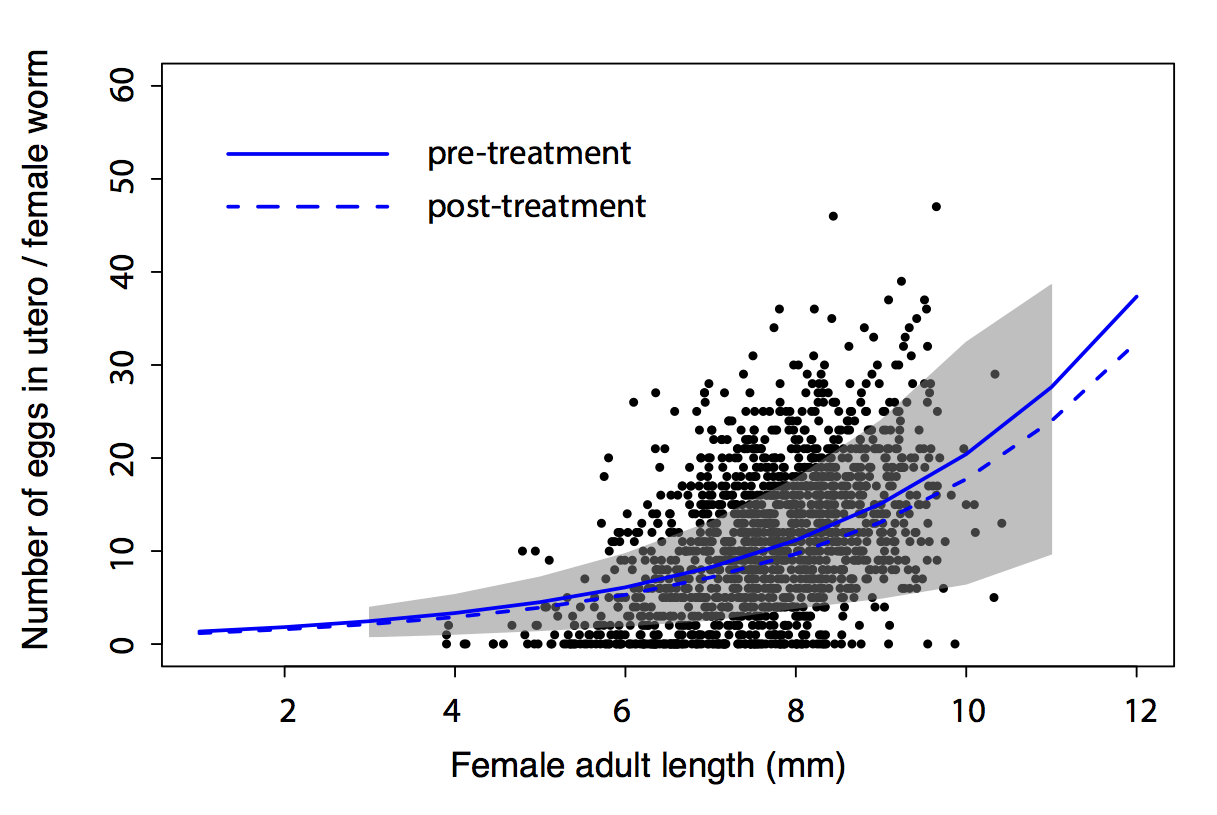

Supplement: S2 Fig — A random number of T. retortaeformis specimens were collected from the small intestine of rabbits at every sampling point and their body length and number of eggs in utero -for the females- were measured using our established protocol [51, 54]. Eggs in utero were positively correlated to female body length (Generalized Linear Mixed model, GLMM, with a log-link function, Poisson error distribution and rabbit as a random effect)(S4 Table), however, this relationship was not significantly different in the pre- and post-anthelminthic treatment phases of the experiment (p = 0.35). Grey shading gives a 95% prediction interval. (TIFF) [file pcbi.1006167.s003.tiff]

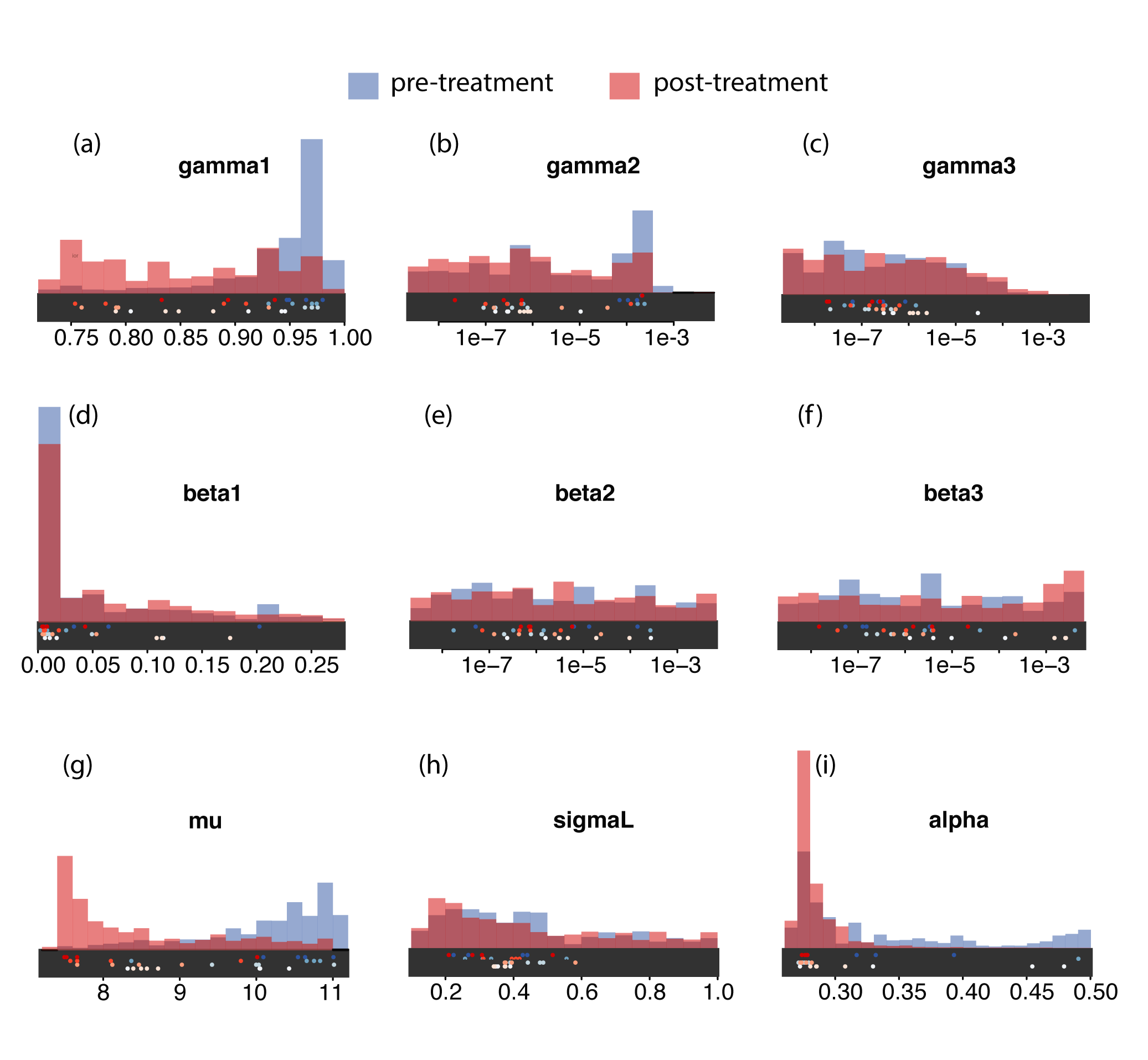

Supplement: S3 Fig — Panels give the sampled posterior distribution, conditional on the observations from all rabbits, for (a) baseline establishment, γ1, (b) effect of cumulative exposure on establishment, γ2, (c) effect of parasite intensity on establishment, γ3, (d) maximum daily clearance, β1, (e) effect of cumulative exposure on establishment, β2, (f) effect of parasite intensity on establishment, β3, (g) mean final length, μL, (h) variance in final parasite length, and σL (i) parasite growth rate, α. The posterior for pre-treatment animals is shown in blue, the posterior for post-treatment animals is shown in red. The x-axis in each panel spans the range of the uniform prior for each parameter. Below each panel is shown the posterior mean (circle) for each individual animal and the central 95% of the rabbit-specific posterior distribution. Estimates for animals sampled on days 15, 30, 45, 60 are plotted from top to bottom; pre-treatment animals are shown in blue, post-treatment animals are shown in red. (TIF) [file pcbi.1006167.s004.tif]

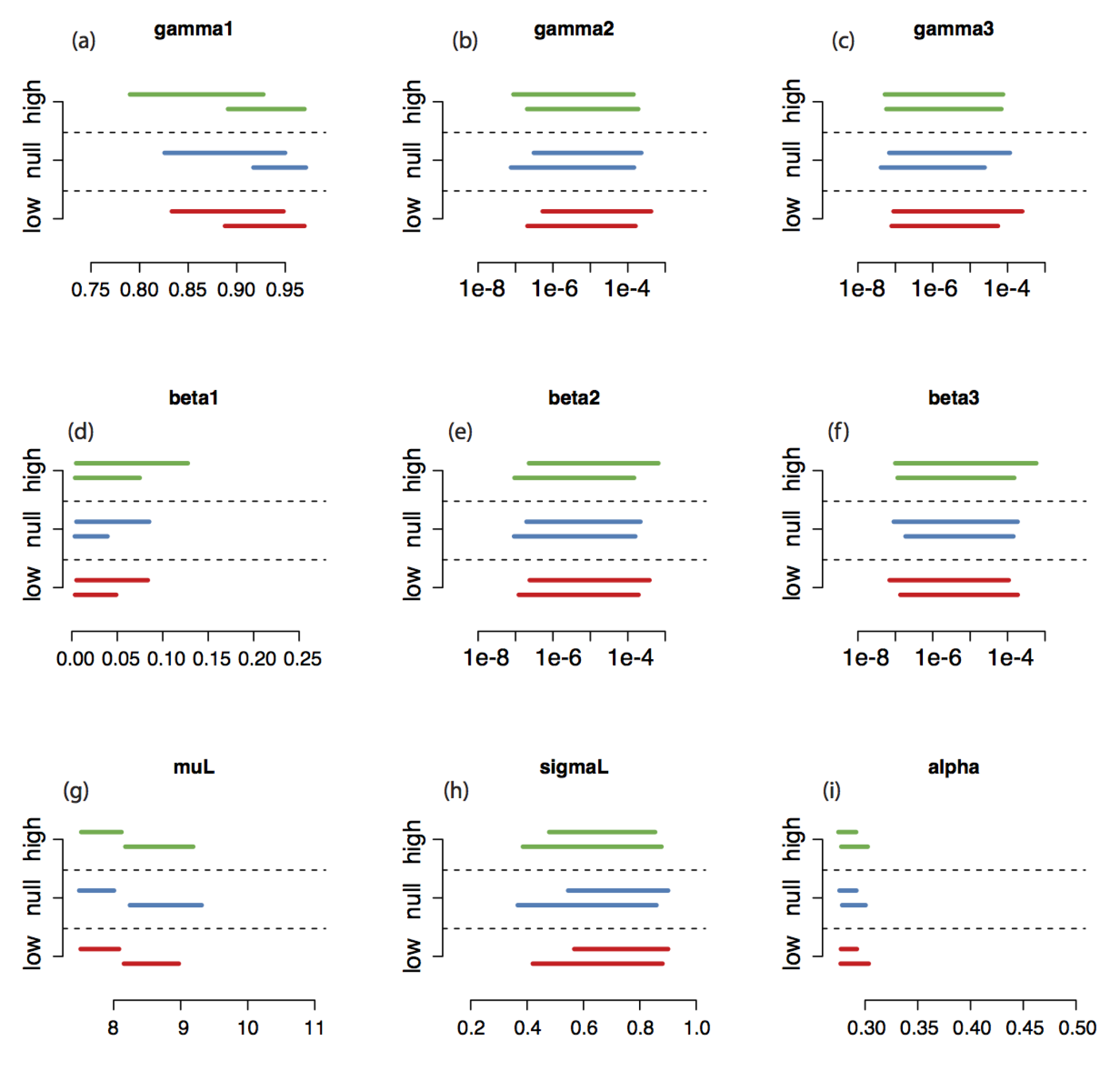

Supplement: S4 Fig — In the main text, we present a model that assumes 3% of eggs shed in the feces are counted, based on 30g dry weight of feces produced per day. Each panel gives the central 95% of the sampled posterior distribution for pre-treatment (lower line) and post-treatment (upper line) animals assuming that observed egg shedding was 50% lower (low), and 50% higher (high) than the assumption made in the main text (null). Individual panels indicate each parameter (a) baseline establishment, γ1, (b) effect of cumulative exposure on establishment, γ2, (c) effect of parasite intensity on establishment, γ3, (d) maximum daily clearance, β1, (e) effect of cumulative exposure on clearance, β2, (f) effect of parasite intensity on clearance, β3, (g) mean final length, μL, (h) variance in final parasite length, and σL (i) parasite growth rate, α. The absence of lines indicates a model for which a given parameter was not present. (TIF) [file pcbi.1006167.s005.tif]

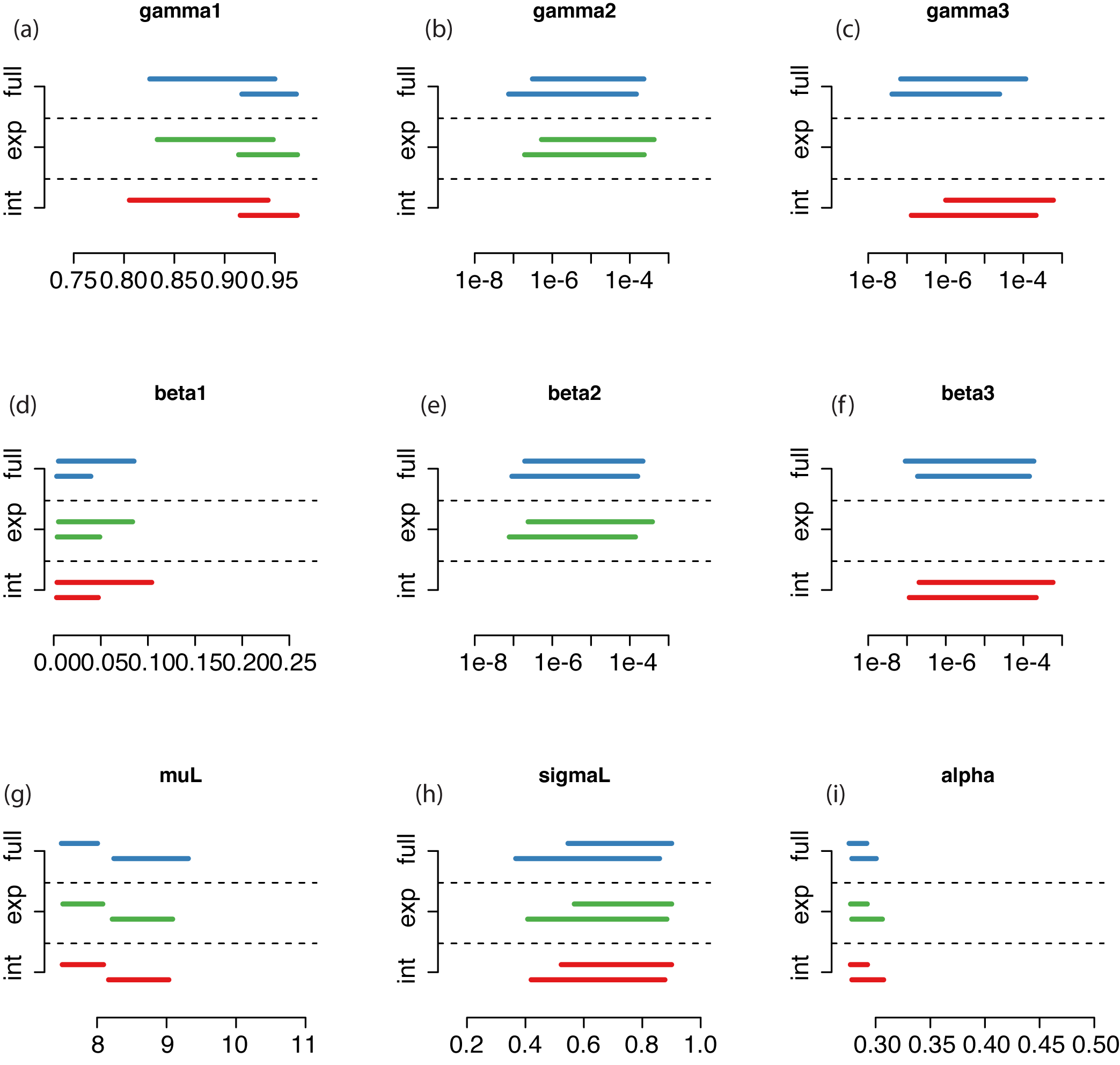

Supplement: S5 Fig — In the main text, we present a model with effects of both cumulative parasite burden and parasite intensity on both larval establishment and adult clearance. Each panel gives the central 95% of the sampled posterior distribution for pre-treatment (lower line) and post-treatment (upper line) animals for the intensity-only (int), exposure-only (exp), and full models (full). Individual panels indicate each parameter (a) baseline establishment, γ1, (b) effect of cumulative exposure on establishment, γ2, (c) effect of parasite intensity on establishment, γ3, (d) maximum daily clearance, β1, (e) effect of cumulative exposure on clearance, β2, (f) effect of parasite intensity on clearance, β3, (g) mean final length, μL, (h) variance in final parasite length, and σL (i) parasite growth rate, α. (TIF) [file pcbi.1006167.s006.tif]
