# Supplementary material for: Changes in parasite traits, rather than intensity, affect the dynamics of infection under external perturbation
Source: PLoS Comput Biol. 2018 Jun 11;14(6):e1006167. doi: 10.1371/journal.pcbi.1006167 (PMC6019670; doi:10.1371/journal.pcbi.1006167)
Supplement: S1 Table — Prior distributions were uniform on a transformed scale. (PDF) [file pcbi.1006167.s007.pdf]

Supporting Table

TableS1: Prior distributions for model parameters. Prior distributions were uniform on a transformed scale.

Parameter	Description	Prior range	Transformation
γ_1	the baseline probability of parasite establishment	(-1,5)	inverse logit
γ_2	the effect of cumulative exposure on parasite establishment	(-20,5)	exponential
γ_3	the effect of adult intensity on parasite establishment	(-20,5)	exponential
β_1	the baseline probability of parasite clearance	(-7,1)	inverse logit
β_2	the effect of cumulative exposure on parasite clearance	(-20,5)	exponential
β_3	the effect of adult intensity on parasite clearance	(-20,5)	exponential
μ_L	the mean final length of adult parasite	(7,11)	none
σ_L	the variance of parasite length at age t days	(-2,0)	exponential
α	parasite growth rate	(-1,0)	inverse logit
