# Supplementary material for: Changes in parasite traits, rather than intensity, affect the dynamics of infection under external perturbation
Source: PLoS Comput Biol. 2018 Jun 11;14(6):e1006167. doi: 10.1371/journal.pcbi.1006167 (PMC6019670; doi:10.1371/journal.pcbi.1006167)
Supplement: S2 Table — The base model includes only the sampling date (DPI), subsequent models include all variables in the rows above. (PDF) [file pcbi.1006167.s008.pdf]

Supporting Table

TableS2: ANOVA table to test alternate models for total number of adult parasites per rabbit. The base model includes only the sampling date (DPI), subsequent models include all variables in the rows above. There was no difference in the total number of worms per rabbit at sacrifice. In the base model, neither linear nor quadratic effects of days post infection on total worms were significant ($p > 0.05$). As noted in the main text, while this is evidence that there is not a strong effect of the experimental phase on worm intensity, direct interpretation of these data to infer within-host population dynamics is flawed as these data do not reflect true time series.

Model	F	p-value(F-test)
Days post infection (DPI)		
+experiment phase	.40	0.52
+experiment phase by DPI interaction	1.24	0.30
