# Supplementary material for: Changes in parasite traits, rather than intensity, affect the dynamics of infection under external perturbation
Source: PLoS Comput Biol. 2018 Jun 11;14(6):e1006167. doi: 10.1371/journal.pcbi.1006167 (PMC6019670; doi:10.1371/journal.pcbi.1006167)
Supplement: S3 Table — The base model includes only the sampling date (week), subsequent models include all variables in the rows above. In all models, the rabbit from which parasites were sampled, was treated as a random effect. (PDF) [file pcbi.1006167.s009.pdf]

Supporting Table

TableS3: Likelihood ratio tests of alternate models for eggs per gram (epg) feces. The base model includes only the sampling date (week), subsequent models include all variables in the rows below. In all models, the rabbit from which worms were sampled, was treated as a random effect. EPG is lower in second phase of the experiment; effect size is -415 (s.e. = 76)

Model	df	Log-likelihood	Likelihood-ratio	p-value(F-test)
Experiment Week	10	-2494		
+experiment phase	11	-2480	27.8	<0.001
+experiment phase by week interaction	18	-2452	56.1	<0.001

Table S3 illustrates that EPG is lower in second phase of the experiment; effect size -415, standard error 76.
