# Supplementary material for: Changes in parasite traits, rather than intensity, affect the dynamics of infection under external perturbation
Source: PLoS Comput Biol. 2018 Jun 11;14(6):e1006167. doi: 10.1371/journal.pcbi.1006167 (PMC6019670; doi:10.1371/journal.pcbi.1006167)
Supplement: S4 Table — The number of eggs per female parasite is modeled as a function of parasite length (mm) and experimental phase (pre- and post-) with a random effect the rabbit from which parasites were sampled. We used a log-link function and Poisson error distribution. (PDF) [file pcbi.1006167.s010.pdf]

Supporting Table

TableS4: Summary results for the GLMM for the relation between eggs in the utero and parasite body length (Fig S2). The number of eggs per female worm is modeled as a function of worm length (mm) and experimental phase (pre- and post-) with a random effect the rabbit from which worms were sampled. We used a log-link function and Poisson error distribution.

	Estimate	Standard-error	z-value	p-value
(intercept)	-0.003668	0.131071	-0.03	0.978
length	0.302004	0.009317	32.41	$< 2e-16^{***}$
phase	-0.141123	0.150537	-0.94	0.349
