# Supplementary material for: Changes in parasite traits, rather than intensity, affect the dynamics of infection under external perturbation
Source: PLoS Comput Biol. 2018 Jun 11;14(6):e1006167. doi: 10.1371/journal.pcbi.1006167 (PMC6019670; doi:10.1371/journal.pcbi.1006167)
Supplement: S5 Table — The base model includes only the sampling date (DPI), subsequent models include all variables in the rows above. In all models, the rabbit from which parasites were sampled was treated as a random effect. (PDF) [file pcbi.1006167.s011.pdf]

Supporting Table

TableS5: Likelihood ratio tests of alternate models of female worm length. The base model includes only the fixed effect for sampling date (DPI), subsequent models include all variables in the rows above. In all models, the rabbit from which worms were sampled was taken was treated as a random effect. Female worms in the post-treatment phase of the experiment were significantly shorter than in the pre-treatment phase; effect size -0.6 mm, standard error 0.04. The linear effect of DPI on worm length was significant (p = 0.08) with effect size -0.34 mm (s.e.=0.19) per 15-day sampling period. We note that the results are similar when male worms are included, but male worms do not shed eggs

.

Model	df	Log-likelihood	Likelihood-ratio	p-value(F-test)
Days post infection (DPI)	6	-2020		
+section of the intestine	7	-1996	48	<0.001
+experiment phase	8	-1990	11	0.006
+experiment phase by DPI interaction	10	-1989	2	0.5
